# A Heating and Cooling Stage With Fast Temporal Control for Biological Applications

**DOI:** 10.1109/OJEMB.2024.3426912

**Published:** 2024-07-12

**Authors:** Manon Valet, Juan M. Iglesias-Artola, Falk Elsner, Anatol W. Fritsch, Otger Campàs

**Affiliations:** ^1^ Cluster of Excellence Physics of LifeTU Dresden 9169 01062 Dresden Germany; ^2^ Max Planck Institute of Molecular Cell Biology and Genetics 28271 01307 Dresden Germany; ^3^ Center for Systems Biology Dresden 01307 Dresden Germany

**Keywords:** Live microscopy, poikilothermy, temperature control, thermal cardiac tolerance, zebrafish

## Abstract

The study of biological processes involving live microscopy techniques requires adequate temperature control to respect the physiology of the organism under study. We present here a design strategy for a microscope temperature stage based on thermoelectric elements. The design allows the user to access a range of temperatures below and above room temperature and can accommodate samples of different geometries. In addition, by cooling simultaneously the sample insert and the objective, we minimize the temperature gradients along the sample for large magnification objectives requiring immersion oil. We illustrate how this design can be used to study the physiology of the zebrafish embryo over the temperature tolerance of this species. We envision that this device could benefit the communities using model and non-model organisms with physiological temperatures different from typical mammalian cell culture incubation in biomedical research.

## I. Introduction

Temperature affects biological processes across all levels of organization [1]. Despite its fundamental role in biology, our knowledge on the effect of temperature shifts on development and physiology is often limited by the inherent difficulties of flexible temperature control on a microscope stage. Among those issues rank temperature inhomogeneities within the sample, slow temperature equilibration, temperature fluctuations and cooling below room temperature.

Several studies have approached these issues in different ways. Sample heating has been achieved in small inserts with heating wires embedded in metal frames [2], or by ensuring a constant supply of warm air [3]. To achieve both heating and cooling capabilities, thermoelectric elements (Peltiers) are used with relatively flat samples such as protein preparations [4], cell culture [5] or small model organisms [6], [7]. Alternatively, liquid-cooled and -heated stages can be used for larger samples with slower equilibration times [8], [9]. Air conditioning systems can also be used to regulate the temperature of the entire imaging room, but are too slow to study the effects of temperature change in the same organism during development [10].

To overcome these challenges, we designed a system that allows temperature control both below and above room temperature - 20$\,^{\circ }$C to 30$\,^{\circ }$C - while minimizing the temperature gradients within the sample, for a zebrafish embryo that can be considered as a relatively large-sized specimen and is an increasingly popular vertebrate model [11]. This system thus facilitates experimental designs studying the physiology of this organism. We present as a short application the temperature-dependence of cardiac rhythm in the zebrafish embryo. We envision that the device could be adapted to other organisms with a wide temperature tolerance range, from marine organisms to many vertebrates.

## II. Results

### A. Stage Design

**Figure 1.**
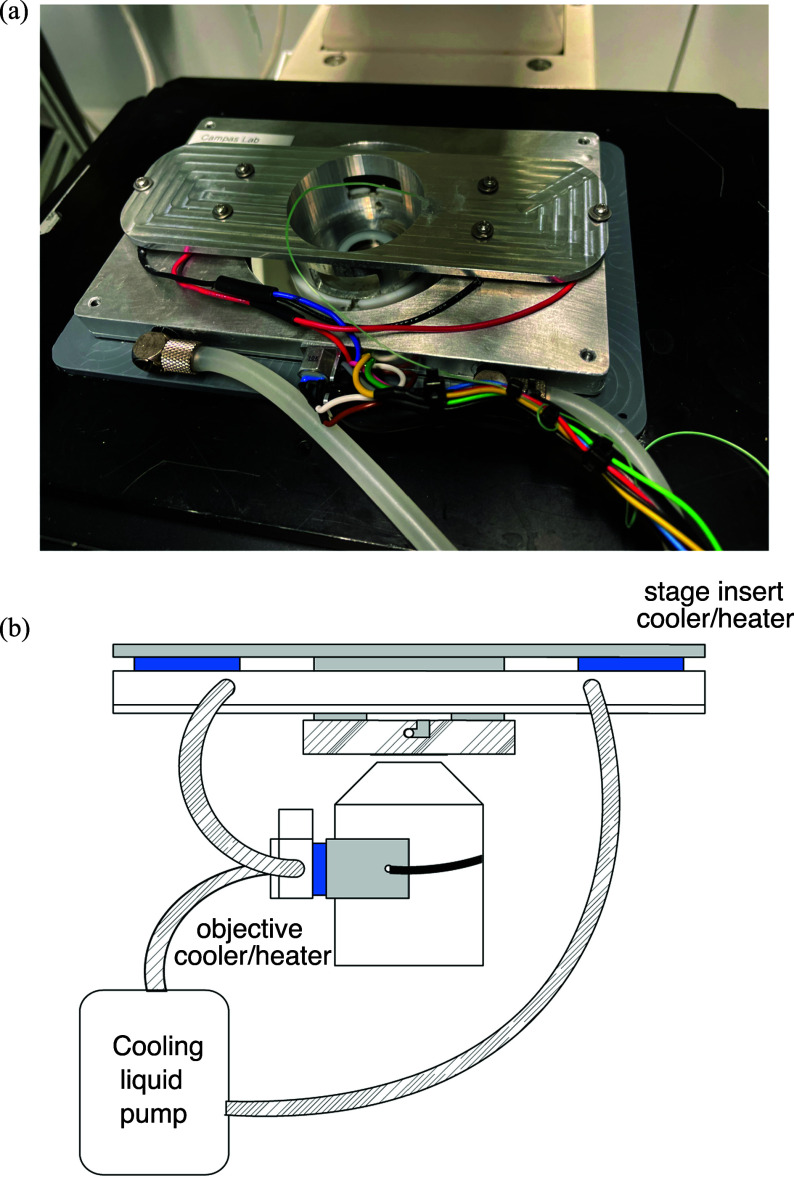
(a) Picture of the stage insert in place on the microscope. (b) Drawing of the connection between the stage insert, the objective cooler/heater and the fluid pump showing the fluid circulation and the whole system assembled.

We develop a new temperature-controlled stage design adapted to zebrafish embryos using a previously published strategy based on thermoelectric elements [7]. The temperature stage consists of two parts: a stage insert and an objective cooler/heater (Fig. 1). The temperature of the stage insert and of the objective cooler/heater is measured separately with two type K thermocouples and can be adjusted with thermoelectric elements (Peltier). To dissipate the heat generated by the Peltier elements, the hot sides were connected to an aluminum heat sink with interior channels for water cooling. An additional NTC thermocouple was used to measure the temperature in the stage insert's heat sink. The Peltier elements are driven by a two channel PID controller, TEC-1122-SV (Meerstetter Engineering). A stage adapter was manufactured to fit the insert on the XY stage of a Zeiss Axiovert. The objective cooler was coupled to a 40X immersion objective.

### B. Technical Performances

After calibrating the device in the center, the precision and accuracy are respectively 0.1$\,^{\circ }$C and 0.5$\,^{\circ }$C for a target temperature of 30$\,^{\circ }$C, and 0.1$\,^{\circ }$C and 0.1$\,^{\circ }$C for a target temperature of 20$\,^{\circ }$C. The device can ramp from 20$\,^{\circ }$C to 30$\,^{\circ }$C in 10 min.

**Figure 2.**
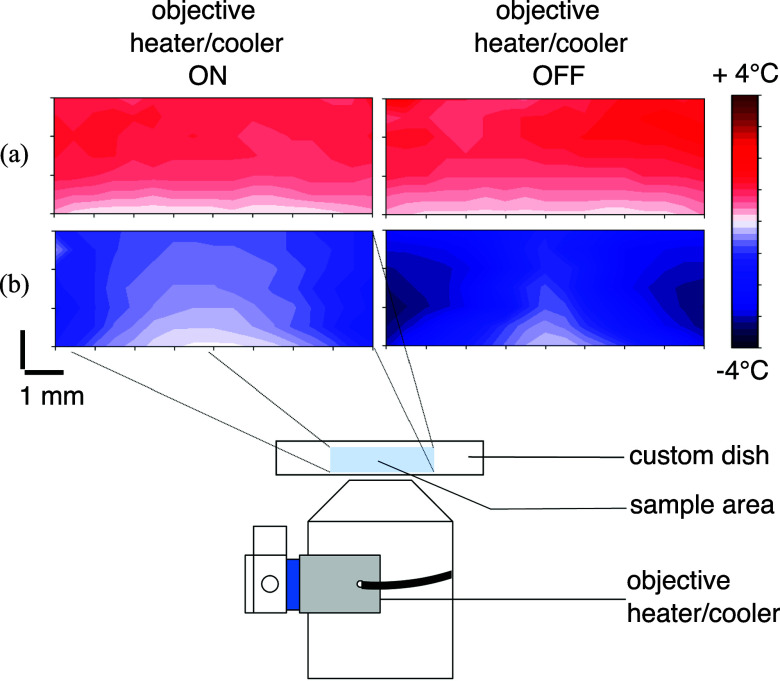
Temperature field in the sample measured with a third thermocouple. Temperature isolines are separated by 0.2$\,^{\circ }$C. (a) Temperature in the top insert is set at 32$\,^{\circ }$C and temperature in the center of the sample is respectively 29$\,^{\circ }$C (objective heater on) and 28$\,^{\circ }$C (objective heater off). (b) Temperature in the top insert is set at 14$\,^{\circ }$C and temperature in the center of the sample is respectively 20.5$\,^{\circ }$C (objective cooler on) and 22.5$\,^{\circ }$C (objective cooler off).

We further characterized the temperature field in the sample using a third probe (Supp.Mat.) for temperatures above and below room temperature. We show that the addition of a cooler ring around the objective diminishes the temperature gradient in the sample along the optical axis and in the imaging plane for temperatures below the room temperature (Fig. 2). With the actual configuration, we achieve temperature gradients lower than 1$\,^{\circ }$C/mm at the center of the stage.

### C. Application to Zebrafish Physiology

**Figure 3.**
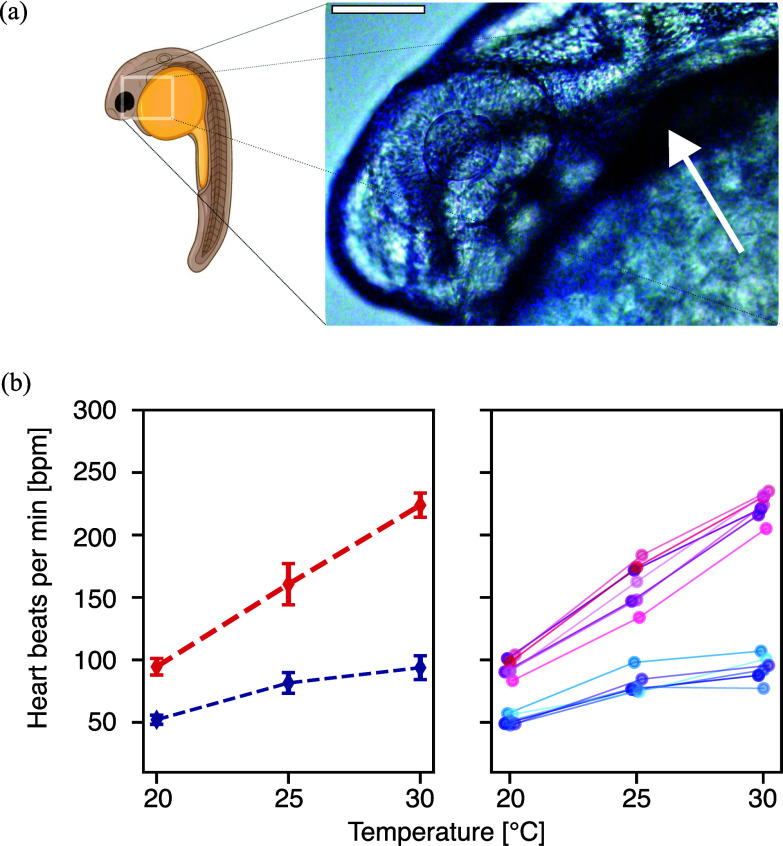
Variation of the heart rate of the zebrafish embryo as a function of temperature. (a) Cartoon of the embryo at 24 hpf with insert representative picture of the heart. Scalebar = $100\:\mathrm{\mu m}$, created with BioRender.com. (b) Heart rate (beats per min or bpm) as a function of the temperature in the sample for two different stages, 24 hpf (blue) and 48 hpf (red). The right panel is showing the trends for each individual embryo.

To demonstrate the applicability of the device in biomedical research, we examined how temperature affects the heart rate of zebrafish embryos at 24 hours and 48 hours post-fertilization (hpf). Prior studies have shown that calcium sensitivity in cardiac filaments is temperature dependent [12], [13] and that the heart rate increases with temperature [14]. We recover the same trend for both embryonic stages (Fig. 3). We can also measure the changes in heartbeat with temperature on the same embryo, as opposed to ensemble averaging. Our values for 48 hpf embryos are in close agreement with previously published findings with relative errors of 14%, 1% and 8% on bpm at 20, 25 and 30$\,^{\circ }$C [14].

## III. Discussion

The portable device presented here allows temperature control on the microscope stage in a physiological range (between 20 and 30$\,^{\circ }$C) while minimizing the temperature gradients within the sample for low temperatures. A key feature of the device is that physiological changes depending on temperature can be tracked individually for biological replicates due to the ability of the device to operate temperature steps within a reasonable time frame.

## IV. Conclusion

Temperature control on the microscope stage can be achieved within a physiological range using thermoelectric elements and heat conductive materials. The thermal gradients in the sample can be minimized using an objective cooler/heater. With the current configuration, we have been able to access temperatures in the 19-37$\,^{\circ }$C range. We encounter some limitations in the lowest accessible temperatures due to the nominal power of the thermoelectric elements. Further optimization on the choice of materials and on the geometry to limit heat loss could marginally improve the functioning of the device towards lower or higher temperatures.

## Materials and Methods

The stage was initially calibrated over the physiological range of the zebrafish embryo with a third thermocouple probe inserted in the sample. Zebrafish experiments were performed following all ethical regulations and according to protocols approved by the European Union (EU) directive 2010/63/EU as well as the German Animal Welfare act.

## Supplementary Materials

Additional details on the electronical and optomechanical elements used for the experiments as well as the complete procedure for tuning the PID and calibrating the stage are provided in the Supplementary Materials. We also detail there zebrafish sample preparation.

Supplementary materials

## Author Contributions

**MV**: Conceptualization, Methodology, Validation, Formal analysis, Investigation, Writing - Original Draft, Writing - Review & Editing. **JMIA**: Conceptualization, Methodology, Investigation, Writing - Original Draft, Writing - Review & Editing, Funding Acquisition. **FE**: Methodology, Investigation, Resources, Writing - Review & Editing, Funding Acquisition. **AWF**: Conceptualization, Methodology, Writing – Review & Editing, Funding Acquisition. **OC**: Conceptualization, Writing – Review & Editing, Supervision, Funding Acquisition.

## Acknowledgment

The authors thank the LMF (MPI-CBG) for the generous loan of a Zeiss Axiovert used in those experiments and the BMS (MPI-CBG) for maintenance and breeding of the fish lines used in this study.

## Conflict of interest

AF, JMIA and FE have filed a patent application for the temperature stage. AF and JMIA are part of MAX!mize, the official start-up incubation program for the Max Planck Society by Max Planck Innovation, to explore the commercial potential of the temperature stage. MV and OC declare no conflicts of interest in this manuscript.
