# Supplementary material for: A Heating and Cooling Stage With Fast Temporal Control for Biological Applications
Source: IEEE Open J Eng Med Biol. 2024 Jul 12;5:573–5. doi: 10.1109/OJEMB.2024.3426912 (PMC11329223; doi:10.1109/OJEMB.2024.3426912)
Supplement: Supplementary materials [file supp1-3426912.pdf]

Supplementary Materials

A heating and cooling stage with fast temporal control for biological applications

Manon Valet[1], Juan M. Iglesias-Artola[2], Falk Elsner[2], Anatol W. Fritsch[2] and Otger Campàs[1,2,3]
[1] Cluster of Excellence Physics of Life, TU Dresden, 01062 Dresden, Germany

[2] Max Planck Institute of Molecular Cell Biology and Genetics, Pfotenhauerstrasse 108, 01307 Dresden, Germany
[3] Center for Systems Biology Dresden, 01307 Dresden, Germany
CORRESPONDING AUTHORS: Manon Valet (e-mail: manon.valet@tu-dresden.de) and Juan M. Iglesias-Artola (e-mail: jiglesia@mpi-cbg.de)

I. General Information

THE temperature on the stage is controlled via a dual-channel PID controller TEC-1122-SV module VIN1 (Fig. 1) equipped with the thermocouple type K readout TCI-1181 (Meerstetter Engineering). This PID controller reads the temperature in the stage insert and the objective cooler/heater via two independent type K thermocouples (5SC-TT-KI-40-2M, Omega). The calibration is done with a third thermocouple in the sample (Fig. 2). The temperature in the heat sink for both constructions is also integrated with two thermistors type NTC (B57045K0103K000, EPCOS). The PID controller modulates the heat transfer to the bridge on the stage insert (Fig. 3) and the objective cooler/heater (Fig. 4) via two thermoelectric elements for the stage insert (ET-127-08-15-RS, Adaptive) and one for the objective heater/cooler (CP0.8-31-06L, Laird Technologies). The cooling system is a water cooler (Alphacool, eiswand 360 CPU). The stage heat sink (Fig. 5) and the objective heat sink (Fig. 4) are connected to the same circulation system (Fig. 1). The sample is mounted in a custom dish that can be fitted easily within the device (Fig. 6). Measurements for the objective cooler/heater were taken for a 40x water immersion objective (Zeiss, Objective LD C-Apochromat 40x NA 1.1).

II. Calibration Procedure

To calibrate the stage, we first proceed by positioning a third probe in the center of the sample mounting zone using a HH306A Omega data logger thermometer and an Omega type K thermocouple 5SC-TT-KI-40-2M calibrated between two points (water/ice mixture and boiling water). The device should be calibrated when installed on a new setup, as the heat transfers between the sample and the cooling and heating modules can change depending on the experimental setup (e.g., imaging hardware or cooling system in the room). To calibrate the device, it is necessary to establish the calibration curve between the sample temperature and the temperature set for the stage insert and objective cooler/heater thermocouples (Fig. 2), as well as to tune the PID parameters of the controller. The calibration curve must be established for the temperature range of the study/experiment and for a given microscope, objective type or immersion oil. A new calibration or a re-calibration also requires to tune the PID parameters for the controller to minimize temperature overshoots or oscillations. To facilitate this task, we provide the parameters that can be used as a first guess for the auto-tuning function of the TEC software (Table I).

(a)
(b)

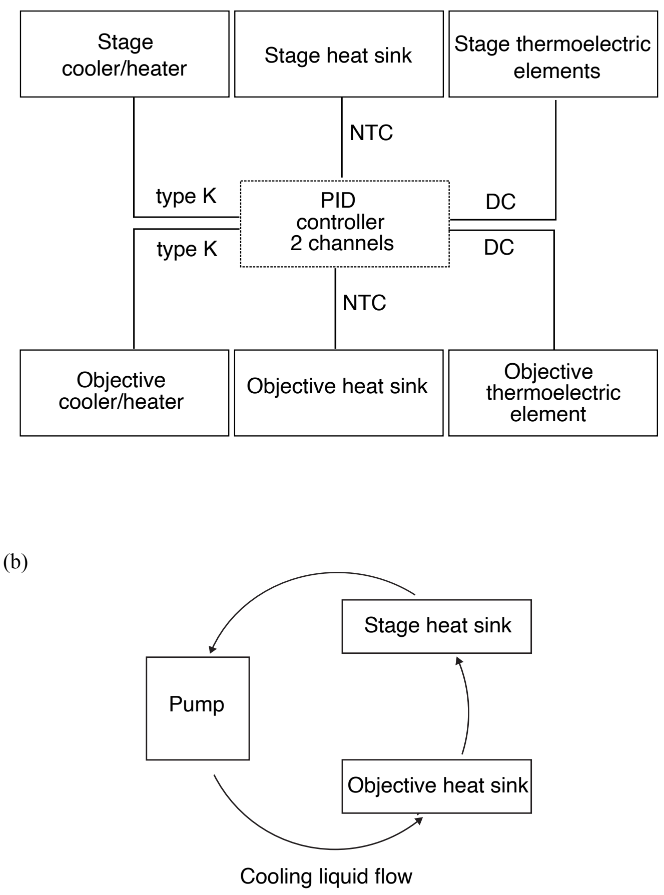

Fig. 1. (a) Diagram showing the connections between the thermocouples, the metal inserts and the thermoelectric elements. (b) Diagram showing the flow circulation between the different elements.

III. Temperature Field

To assess the temperature gradients which may exist in the sample, we mount a third probe connected to the data logger

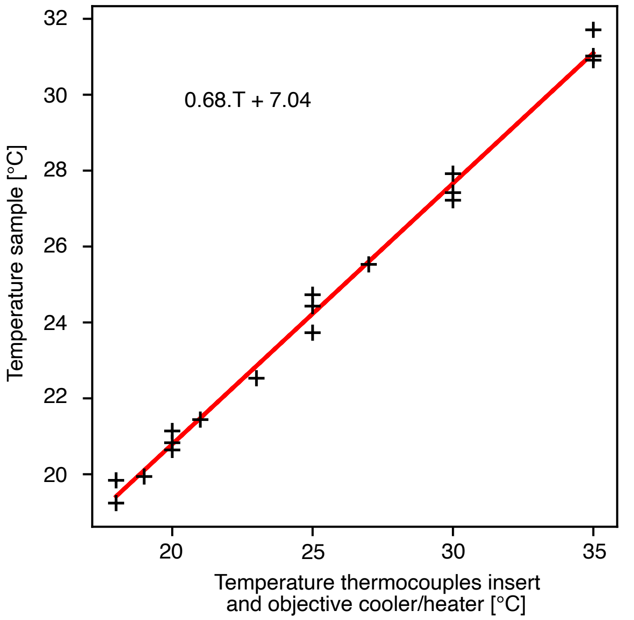

Fig. 2. Representative calibration curve. The temperature of the insert and objective cooler/heater is set in the PID controls. The temperature of the sample is measured with a third probe.

on a three-axis manual micrometer. We take temperature points by 0.5 mm increments in the (xy) plane - or imaging plane - and by 0.5 mm increments along the optical axis (z).

IV. Zebrafish Husbandry and Experimentation

Zebrafish (Danio rerio) were maintained as previously described [1]. Experiments were performed following all ethical regulations and according to protocols approved by the European Union (EU) directive 2010/63/EU as well as the German Animal Welfare act. Embryos were collected immediately post-fertilization and left at 28°C until the time of the experiment (24- or 48-hours post-fertilization).

The embryos were manually dechorionated and anesthesia was performed with MS-222 [100 mg/L] immediately prior to mounting in agar 0.8%.

Imaging was performed with an Axiovert 200 equipped with a Bresser MikroCam II 0.4 UHSP. Heart beats were counted manually with a cell counter for a time span of 5 min for each individual and associated temperature point. The average over 5 min yields the individual heartbeat per minute (bpm) for each specimen.

V. Perspectives and Limitations

We have described a setup designed to study the thermal tolerance of a physiological process (heart rate) in a zebrafish model. With the components we refer here, we have been able to reach temperatures going from 19°C to 37°C, which covers the physiological range of many model and non-model organisms (including, but not limited to, mouse, organoids or amphibians). The performance of the device is mostly determined by the power of the thermoelectric elements (20.9 W for the stage insert and 4.9 W for the objective cooler/heater). More powerful elements can be used if the temperature range to be explored is wider. A possible alternative combination would be a Peltier Module of 40W by CUI Devices (CP402533) for the stage insert, and a 20W Peltier module from Laird Thermal Systems (387005673) for the objective cooler/heater. Additionally, the user can insert an insulating ring between the microscope body and the objective to prevent a thermal connection.

PID PARAMETERS

	Stage Insert	Objective cooler/heater
Coarse Temp Ramp [°C]	0.20	0.04
Proximity Width	14.3	5.24
Kp [%/°C]	24.0	65.6
Ti [s]	30	51.6
Ts [s]	0	0
D	0.3	0.025

TABLE I. PID parameters for both channels of the controller, for a tuning performed after calibration.

References

[1] C. Nusslein-Volhard and R. Dahm, ”Keeping and Raising Zebrafish” in *Zebrafish*, Oxford University Press, 2002, ch. *1*, pp. *7-37*.

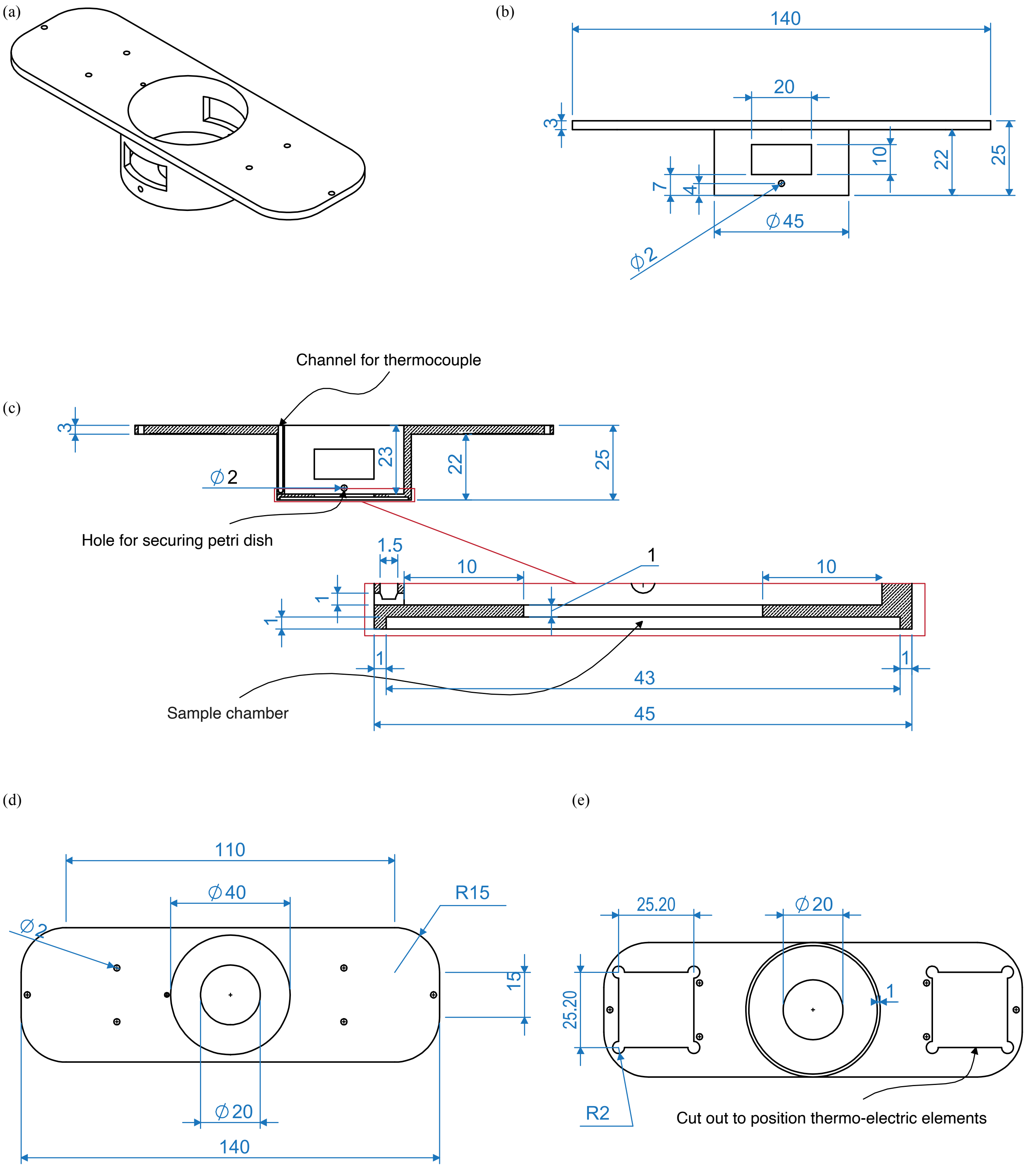

Fig. 3. Aluminum bridge on the stage insert (a) perspective view (b) external side view with dimensions (c) internal side view with dimensions (d) top view with dimensions (e) top view, flipped with the machined cut out to position thermoelectric elements.

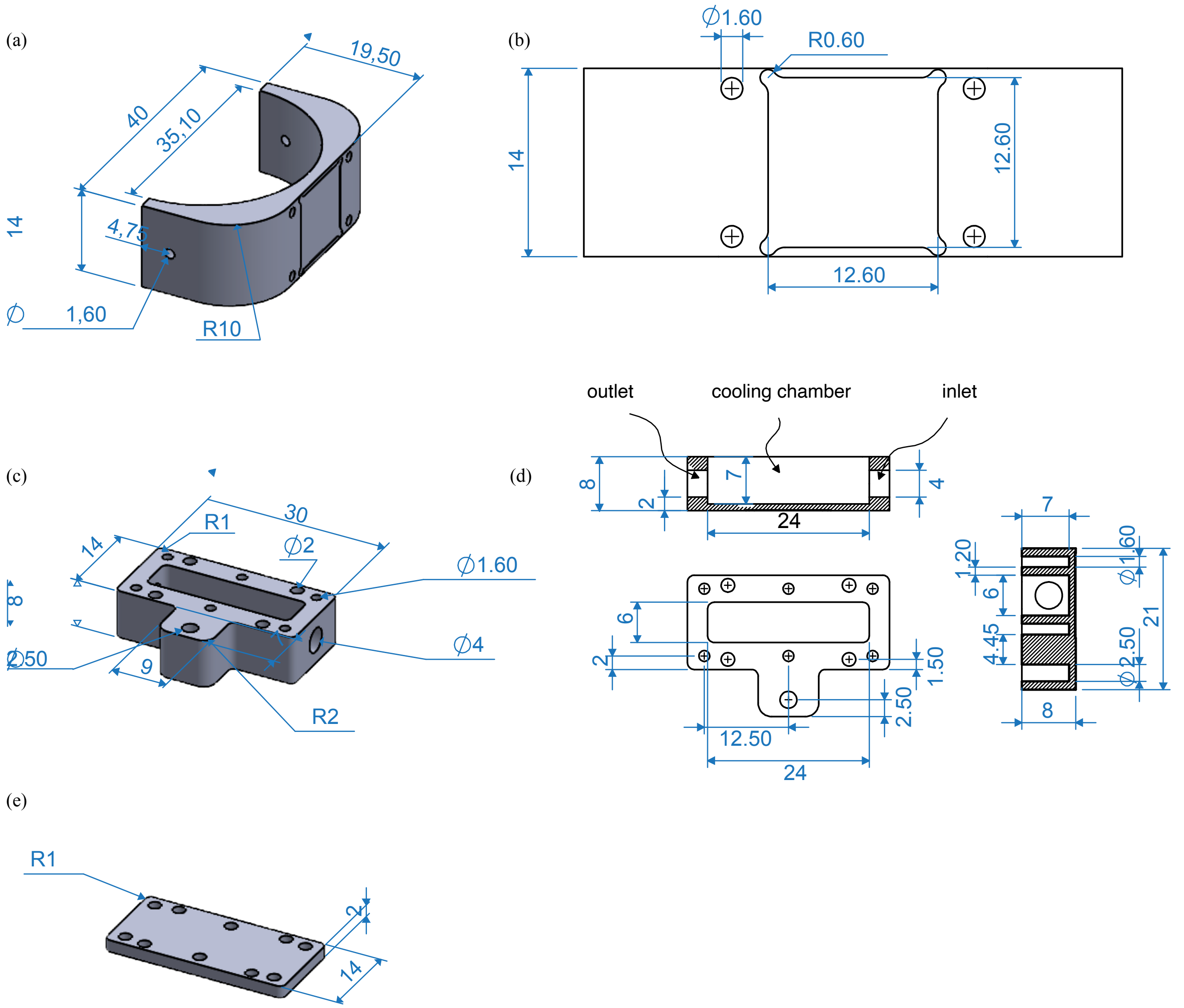

Fig. 4. Objective cooler/heater (a) perspective view (b) side view with machined cut-out for the thermoelectric element (c) perspective view of the associated heat sink (d) inside view of the heat sink with water circulation (e) fixation part for the heat sink.

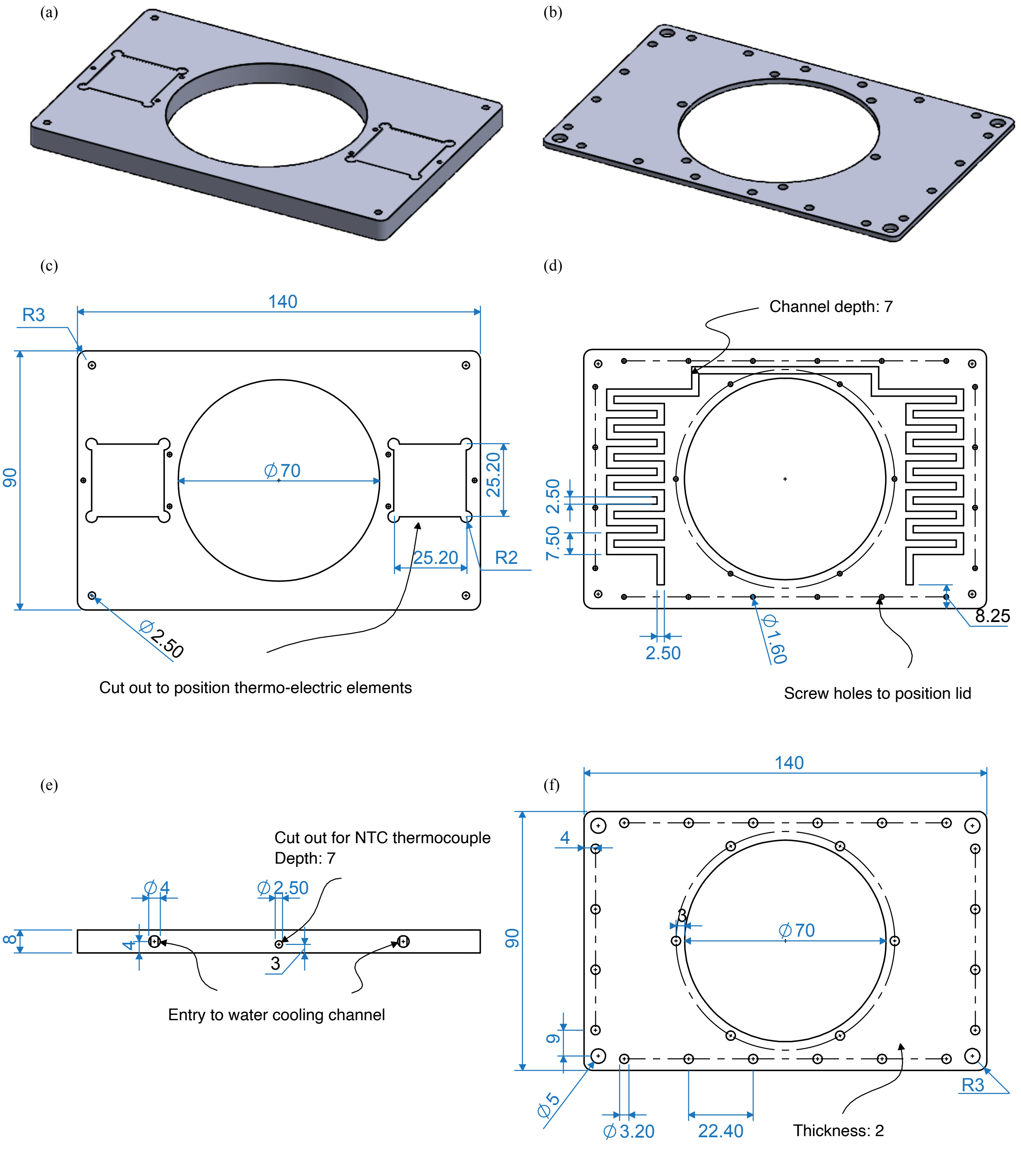

Fig. 5. Heat sink of the stage insert (a) perspective view of the top part (b) perspective view of the bottom part (c) inside view of the top part on the side where the thermoelectric elements are fixed (d) inside view of the top part on the other side where the water channel runs (e) side view of the top part showing entry points for the cooling fluid (f) inside view of the bottom part with fixation points.

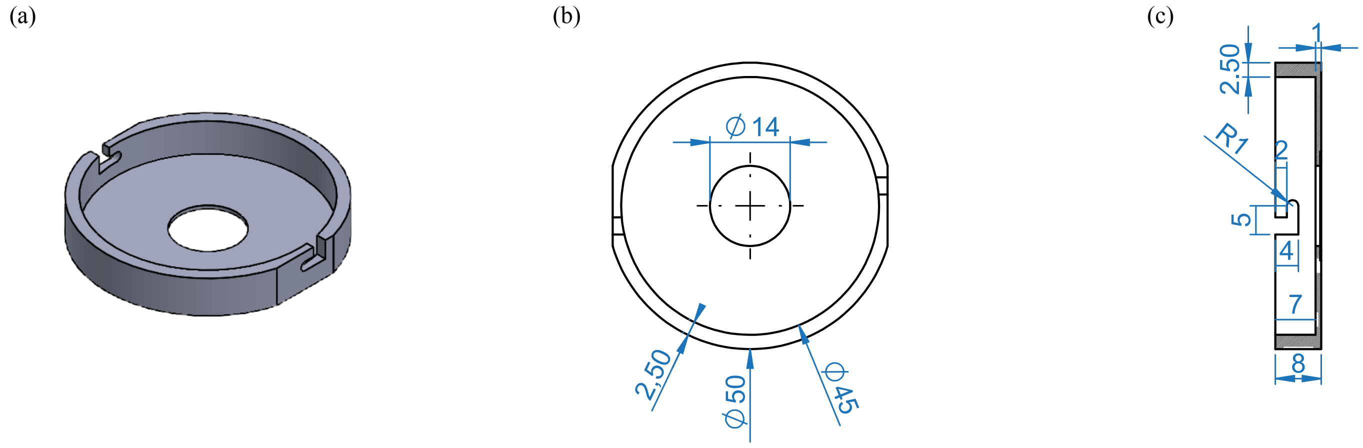

Fig. 6. Sample mounting (a) perspective view (b) inside dimensions, top view (c) inside dimensions, side view.
